# Aquaporin 4 modulation drives amyloid burden and cognitive abilities in an APPPS1 mouse model of Alzheimer's disease

**DOI:** 10.1002/alz.70164

**Published:** 2025-05-06

**Authors:** Marina Daniela Manescu, Bogdan Catalin, Ioana Baldea, Valentin Octavian Mateescu, Gabriela Camelia Rosu, Ianis Kevyn Stefan Boboc, Anca‐Maria Istrate‐Ofiteru, Ilona Mihaela Liliac, Costin Teodor Streba, Samir Kumar‐Singh, Daniel Pirici

**Affiliations:** ^1^ Department of Histology University of Medicine and Pharmacy of Craiova Craiova Romania; ^2^ Department of Physiology University of Medicine and Pharmacy of Craiova Craiova Romania; ^3^ Department of Physiology Iuliu Haţieganu University of Medicine and Pharmacy Cluj‐Napoca Romania; ^4^ Department of Pulmonology University of Medicine and Pharmacy of Craiova Craiova Romania; ^5^ Laboratory of Cell Biology and Histology, Molecular Pathology Group, Faculty of Medical and Health Sciences University of Antwerp Antwerp Belgium

**Keywords:** Alzheimer's disease, amyloid plaques, APPPS1 mouse model, aquaporin‐4, intramural periarterial drainage

## Abstract

**INTRODUCTION:**

Deficiency in the aquaporin‐4 (AQP4) water channel has been linked to impaired amyloid beta (Aβ) clearance. However, a detailed morphopathological analysis of amyloid deposition following AQP4 therapeutic modulation remains unexplored.

**METHODS:**

Two‐month‐old amyloid precursor protein presenilin 1 (APPPS1) mice were treated daily for 28 days with either the AQP4 facilitator N‐(3‐(Benzyloxy)pyridin‐2‐yl) benzene‐sulfonamide (TGN‐073) or the AQP4 inhibitor N‐(1,3,4‐thiadiazol‐2‐yl)pyridine‐3‐carboxamide dihydrochloride (TGN‐020) (both at 200 mg/kg). Controls included vehicle‐treated APPPS1 and WT C57BL/6J mice. Comprehensive histopathological, biochemical, and behavioral analyses were conducted.

**RESULTS:**

Mice treated with AQP4 facilitator showed a significant reduction in total Aβ, fibrillar deposits, and soluble Aβ, while the AQP4 inhibitor caused a substantial increase in brain Aβ. AQP4‐facilitator treatment also reduced Aβ40 levels and Aβ40/Aβ42 ratio, whereas the inhibitor treatment increased both Aβ40 and Aβ42. Additionally, facilitator‐treated mice demonstrated reduced anxiety and improved memory performance.

**DISCUSSION:**

These findings suggest that AQP4 modulation is a promising strategy to enhance Aβ clearance and a potential therapeutic target in Alzheimer's disease.

**Highlights:**

- Intramural periarterial drainage of the interstitial fluid mediated by aquaporin‐4 (AQP4) is a key element that ensures clearance of catabolites/Aβ peptide from within the brain parenchyma.
- Inhibition of AQP4 in an APPPS1 mouse model of AD leads to increased amyloid deposition and deficient behavior compared to untreated transgenic animals.
- Pharmaceutical facilitation of AQP4 in the same APPPS1 mouse model leads to a massive decrease in amyloid burden and improves the behavioral performance of the animals compared to untreated control APPPS1 mice.

## Graphical abstract

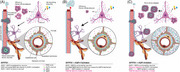

## 1 BACKGROUND

The histopathology of Alzheimer's disease (AD), the most important form of dementia in all age groups, is centered around the accumulation of amyloid beta peptide (Aβ) plaques and neurofibrillary tangles in the brains of patients.^1^, ^2^, ^3^ Causative gene mutations have been associated with autosomal dominant familial AD (FAD), such as amyloid precursor protein (*APP*) and presenilin 1 and 2 (*PS1, PS2*), and genetic risk factors such as Apolipoprotein E (*APOE*), sortilin‐related receptor with type‐A repeats (*SORL1*), and so forth; however, FAD accounts for less than 5% of all AD cases.^4^, ^5^, ^6^, ^7^ Advanced age is the most important known risk factor for AD, and sporadic late‐onset AD (LOAD) cases account for the majority of patients, suggesting that age‐related loss of function of clearance pathways might be more central to the disease rather than Aβ overproduction alone.^8^, ^9^ Despite many mechanisms governing the toxicity of Aβ being described, such as the direct toxicity, metabolic dysfunction, and imbalances of oxidative stress pathways,^8^, ^9^, ^10^ it seems that oligomers/protofibrils and insoluble species' fibrils are mostly responsible for Aβ toxicity.^10^, ^11^, ^12^, ^13^ Thus, distinct pathological roles have been attributed to soluble and insoluble Aβ fractions, with high levels of soluble Aβ40 isoforms accumulating in/around the blood vessel walls as cerebral amyloid angiopathy (CAA), dysphoric angiopathy, and vasculotropic amyloid plaques, which are related directly to patient cognitive decline.^14^, ^15^, ^16^, ^17^ While most soluble Aβ40 is cleared across perivascular spaces together with the interstitial fluid (ISF) through the so‐called intramural periarterial drainage (IPAD) pathway, it is of great interest to describe the mechanisms that govern Aβ clearing at the level of perivascular glia limitans and the extent to which modulating these mechanisms might serve as a putative therapeutic approach to compensate their loss of function that inherently occurs with advancing age.^18^, ^19^, ^20^

Aquaporin‐4 (AQP4), the most abundant water channel in the brain, acts at the level of the perivascular astrocyte endfeet as a bidirectional osmosis‐driven gateway, controlling both the influx of cerebrospinal fluid (CSF) toward the neuropil, and the ISF efflux into the perivascular spaces that drain to dural arachnoid granulations and to lymphatics of the nose and neck.^18^, ^21^, ^22^ Previous studies using AQP4 KO or overexpressing mouse models or pharmacological agents such as the AQP4 inhibitor N‐(1,3,4‐thiadiazol‐2‐yl)pyridine‐3‐carboxamide dihydrochloride (TGN‐020) and the facilitator N‐(3‐(Benzyloxy)pyridin‐2‐yl) benzene‐sulfonamide (TGN‐073), showed that AQP4 modulation effectively regulated brain water content under various conditions.^23^, ^24^, ^25^, ^26^, ^27^, ^28^, ^29^, ^30^, ^31^, ^32^, ^33^, ^34^, ^35^ Thus, AQP4 KO mice with vasogenic brain edema consecutive to inflammatory obstructive hydrocephalus and brain abscess show increased brain water content, higher intracranial pressure, and worse clinical outcomes compared to WT animals.^23^, ^24^, ^25^, ^26^ Consistent with the hypothesis that AQP4 drives water and small‐molecule influx and efflux around the blood–brain barrier (BBB), intracortical injection of fluorescently labeled soluble Aβ40 in mice showed passage and clearance through perivascular spaces, and with TGN‐020‐mediated AQP4 inhibition, Aβ40 was cleared significantly slower than for the untreated animals and accumulated in vessel walls surrounding the injection site.^30^, ^31^, ^32^ Also, as expected, mice treated with the TGN‐073 AQP4 facilitator showed increased turnover of ISF and increased fluid diffusion in their brains compared to the untreated group.^33^, ^34^ Given both the predicted and observed modulatory effects of AQP4 inhibition and facilitation on perivascular ISF drainage, it is crucial to investigate whether this influence also extends to the clearance of soluble Aβ, to what extent, and how it relates to the turnover of insoluble Aβ species (such as Aβ42) and amyloid plaque formation in Aβ‐overexpressing mouse models.

To gain a deeper understanding of AQP4's role in Aβ clearance under pathological conditions, in this study we assessed the effects of both AQP4 pharmacological facilitation and inhibition in the APPPS1 mouse model. Our aim was to characterize the morphological and semiquantitative changes in amyloid deposits and their impact on mouse behavior.

## 2 METHODS

### 2.1 Animals and treatment

The study was performed on C57BL/6J‐TgN mice carrying mutant human APP (Thy1‐APPKM670/671NL) and mutant human presenilin (Thy1‐PS1L166P)^36^, ^37^ (*N* = 45) and C57BL/6J WT (*N* = 12) mice 2 months of age (Figure S1). Animals were housed in a controlled 12 h/12 h light/dark cycle and fed ad libitum. All experiments were conducted in accordance with the Federation of European Laboratory Animal Science Associations (FELASA), following Declaration of Helsinki ethical guidelines for animal experimentation. The study was approved by Ethical Committee for Animal Experimentation of the University of Medicine and Pharmacy of Craiova (no 93/16.05.2022).

APPPS1 mice were randomized to receive daily i.p. injections of 200 mg/kg of TGN‐020 AQP4 inhibitor (*N* = 15; APPPS1+TGN‐020 group), 200 mg/kg TGN‐073 AQP4 facilitator (*N* = 15; APPPS1+TGN‐073 group), or sterile distilled water (*N* = 15; APPPS1‐sham group). Furthermore, a WT C57BL/6J control group consisted of mice (*N* = 12) also injected with sterile distilled water. Briefly, 20 mg/ml salt solutions of TGN‐020 [N‐(1,3,4‐thiadiazol‐2‐yl)pyridine‐3‐carboxamide dihydrochloride, *M* = 279.1463] and TGN‐073 [N‐(3‐(Benzyloxy)pyridin‐2‐yl benzene‐sulfonamide, *M* = 340.40; Ukrorgsyntez Ltd., Kiev, Ukraine] were freshly prepared with sterile distilled water and titrated with 2 M NaOH to a pH of 8.0. The corresponding treatment was administered in all animal groups i.p. for 28 days. At the end of treatment, animals were euthanized by first anesthetizing those with a mixture of ketamine (120 mg/kg) and xylazine (10 mg/kg) and then performing transcardial perfusion with ice cold 0.9% NaCl, followed by 4% paraformaldehyde (PFA) through a left ventricular catheter till the heart stopped beating.^38^, ^39^

### 2.2 Behavioral testing

An investigator‐blinded behavior test was performed on all mice before the start of the experiment (week 0) and at 2  and 4 weeks prior to organ collection (Figure S1). To reduce the interference between tests, an open field (OF) test was conducted first following the next day by a novel object recognition (NOR) test. After each trial, all surfaces were wiped with 75% ethanol in order to remove any odors. The OF test was performed as previously described.^40^, ^41^ Briefly, mice were placed in the center of a 50 × 33 × 15 cm (L × B × H) OF maze and were allowed to move and explore the arena for 10 min. The behavior of each animal was recorded and automatically analyzed using EthoVision XT 16 (Noldus Information Technology BV, Wageningen, Netherlands). To quantify anxiety and exploration, the time spent in the center arena was recorded and processed.

The NOR test was used to evaluate short‐term memory and performed as previously described.^41^, ^42^ At first, animals were placed in an open arena along with two identical objects situated 15 cm from the side walls in two opposite parts of the maze. Animals were left to freely explore the arena and the object for 6 min before being placed back in their cage. After 60 min, a novel object was placed instead of one of the two identical initial objects. The animals were then given an additional 6 min to explore the new arena. For each animal, the discrimination index (the percentage of the time exploring the novel object among the total time spent exploring both objects) was determined.^43^

**RESEARCH IN CONTEXT**

1. **Systematic review**: A literature review (PubMed) revealed the existence of only a few published studies addressing the influence of AQP4 modulation on amyloid plaque formation in amyloidogenic transgenic mouse models. Moreover, no study is available to look at both AQP4 facilitation and inhibition on the same transgenic background and to analyze the morphology and quantity of amyloid burden.
2. **Interpretation**: Our study reveals profound bidirectional modulation of amyloid deposits following pharmacological AQP4 facilitation and inhibition, as well as of functional memory and anxiety performances of these animals.
3. **Future directions**: Our findings support the potential of AQP4 facilitation strategies in AD therapeutics.

### 2.3 Tissue processing and histopathology

After euthanasia and transcardial perfusion (see preceding discussion), brains were collected and further fixed for 2 days in 4% PFA at room temperature. Hemispheres cut medio‐sagittally were processed for paraffin embedding, and 5‐µm‐thick serial sections were prepared on a rotary microtome HM355S with a waterfall‐based section‐transfer system and a Peltier cooling element (Thermo Fisher Scientific, Walldorf, Germany). A section from each animal series was first prepared and analyzed following routine hematoxylin and eosin (H&E) staining, with the consecutive sections processed for immunohistochemistry.

### 2.4 Immunohistochemistry and thioflavin S staining

Double enzymatic sequential immunohistochemistry was performed to visualize total deposited amyloid burden and the relationship between Aβ deposits and the vasculature. A protocol was first developed for double antigen retrieval procedure in order to ensure the best binding conditions for both antibodies. Briefly, the sections were deparaffinized in xylene, rehydrated in graded alcohol series, processed for the first antigen retrieval by microwaving in 0.1 M citrate buffer pH 6 for 20 min and then further incubated in 70% formic acid for 5 min. After thorough washing in tap water, distilled water, and PBS, the slides were first incubated for 30 min in a 1% hydrogen peroxide solution, blocked for 30 min in 3% skim milk (Bio‐Rad, Munich, Germany), and then incubated with the first primary antibody against endothelia (rat anti‐CD31, 1:50; Biozol Diagnostica Vertrieb GmbH, Hamburg, Germany, product DIA‐310‐BA‐2) for 18 h at 4°C. The next day the signal was amplified for 60 min utilizing an anti‐rat alkaline phosphatase‐conjugated polymer link (Nichirei‐Bioscience, Tokyo, Japan) and detected with a Fast Red formulation substrate (ImmPACT Vector Red, Vector Laboratories, Burlingame, CA, USA). After extended washing in PBS, the slides were incubated with the second primary antibody, a mouse anti‐human Aβ recognizing Aβ40 and Aβ42 forms (4G8 clone, 1:30.000; Merck Chemicals GmbH, Darmstadt, Germany, product MAB1561) for an additional 18 h at 4°C. The signal was amplified for 60 min utilizing an anti‐mouse peroxidase‐conjugated polymer link (Vector Laboratories) and detected with 3′,3′‐diaminobenzidine (ImmPACT DAB, Vector Laboratories). Slides were counterstained with hematoxylin and coverslipped with a glycerol‐based mounting medium (Dako, Glostrup, Denmark).

For fluorescence double immunohistochemistry for Aβ40 and Aβ42, the slides were processed only for formic acid antigen retrieval and incubated overnight at 4°C with a mixture of rabbit anti‐Aβ40 (1:1.000, Thermo Fisher Scientific, Waltham, MA, USA, product PA3‐16760) and of mouse anti‐Aβ42 (1:1.000, GeneTex, Irvine, CA, USA, product GTX635160) primary antibodies. After thorough washing, the signals were simultaneously detected with a mix of anti‐mouse Alexa Fluor 594 and anti‐rabbit Alexa Fluor 488 secondary antibodies (Thermo Fisher Scientific, Waltham, MA USA; 1:300) for 2 h at room temperature and then coverslipped with an antifade mounting medium containing DAPI (Vectashield, Vector Laboratories).

To evaluate the fibrillar Aβ fraction, a consecutive series of slides was processed for ThS staining. Briefly, the sections were deparaffinized in xylene, rehydrated in distilled water, and incubated for 5 min in a filtered 1% aquaeous ThS solution (Sigma‐Aldrich Chemie GmbH, Taufkirchen, Germany). Thereafter, the slides were differentiated for 5 min in 70% ethanol, rinsed in distilled water, and mounted with DAPI‐containing Vectashield.

### 2.5 Microscopic image analysis

For transmitted light microscopy analysis, whole‐brain slides double stained for CD31 and total Aβ were scanned at 80× magnification utilizing a Motic EasyScan Pro 6 slide scanner (Motic Digital Pathology, Barcelona, Spain). The scans were saved in the proprietary pyramidal tiled image format, and all identifiable amyloid deposits were counted and individually measured for their maximum diameter and relative distances to the nearest blood vessels, utilizing the Motic DSAssistant software package. A blood vessel running through or in direct contact with an amyloid deposit was deemed to be at a 0 µm distance from that deposit. Four distinct anatomical areas on sagittal sections were analyzed, comprising neocortex, hippocampus, striatum, and thalamus.^44^

For further Aβ area and expression intensity analysis, whole slides were exported as tiff files and further evaluated in the Image ProPlus 11 image analysis package (Media Cybernetics, Bethesda, MD, USA). Amyloid deposits were next segmented in these images based on their specific color‐intensity histogram pattern, which was defined at the beginning of the analysis by sampling the RGB profile of DAB, with the same threshold pattern being constantly applied on all images. Plaques were segmented as regions of interest (ROIs), for which we calculated areas, integrated optical density (IOD) of the signal (defined as the area × average pixel intensity), and fractal dimension (FD) as a measure of their complex and irregular morphology. FDs were calculated by a box‐counting algorithm as the slope of the regression line for the log‐log plot of the counting box size and the number of counts in Image ProPlus.

High‐resolution (40× [NA = 0.95] and 60× [NA = 1.27]) fluorescence images of all identifiable Aβ40/Aβ42‐positive amyloid depositions within the four considered anatomical regions were acquired utilizing a Nikon AX R inverted confocal microscope built on the Eclipse Ti2 platform, equipped with galvanometric and resonant scanners, a six‐wavelength laser source (405/445/488/514/561/640 nm), and detectors with freely tunable emission bands with ±1 nm accuracy (Nikon Europe BV, Amsterdam, the Netherlands), with acquisition setup conditions being maintained constant through all the experiments. Image grabbing and analysis were based on the Nikon NIS‐Elements Advanced Research and Image ProPlus 11 image analysis packages running on a dedicated Dell Precision Tower 7910 graphic station (2× Intel Xeon 8‐Core E5‐2630 version 3 2.40 GHz, 64GB DDR4, nVidia Quadro M4000 8GB GDDR5). Basically, archived multichannel image files were used to calculate expression areas of the Aβ40 and Aβ42 isoforms, together with the colocalization ratio between the two respective fluorescence channels, which were reported as a two‐color overlapping coefficient (Image ProPlus).

For the ThS‐stained sections, whole slides were scanned using a 20× objective on the motorized platform of the Nikon Eclipse Ti2 microscope in a fluorescence configuration. The CoolLED pE‐800 light source (CoolLED Ltd., Andover, UK), along with dedicated DAPI and Alexa Fluor 488 fluorescence filter sets, was used, and images were captured with a Nikon Digital Sight 10 CMOS 23.9 Mp camera.

Diameters, areas, distances, FD values, Aβ40/Aβ42 area ratio, Aβ40 and Aβ42 overlapping coefficients, and ThS signal areas were averaged for each animal and anatomical region, then averaged for each treatment group. Similarly, IOD values were cumulated for each region and animal, then averaged again for each treatment group. All numerical data were collected and visualized in Microsoft Office Excel 2016 (Microsoft, Redmond, WA, USA) and further analyzed using GraphPad Prism 9.2 (GraphPad Software, Boston, MA, USA). Comparisons were then made for the three pathological groups, namely, the untreated, facilitator‐treated, and inhibitor‐treated APPPS1 groups. To assess statistical differences between untreated and treated APPPS1 groups, a one‐way ANOVA followed by a post hoc Fisher's least significant difference (LSD) test was used for comparing the means of the three groups. Correlations were evaluated using Pearson's correlation coefficient, and differences in the magnitude of these relationships were assessed using a one‐tailed Fisher's *Z* test for independent correlations.^45^ Data are reported as means ± standard error of the mean (SEM). In all cases, *p* < 0.05 was used to indicate statistical significance. Image collages were prepared and annotated utilizing CorelDRAW Graphics Suite 2017 (Corel, Ottawa, Canada).

### 2.6 Preparation of tissue lysates and Western blotting

For the measurement of the soluble Aβ fraction, a commercial formalin‐fixed paraffin‐embedded (FFPE) protein extraction kit was used (Qproteome FFPE Tissue Kit, Qiagen, Hilden, Germany). Briefly, five 10‐µm‐thick serial FFPE sections were cut and collected on non‐adhesive glass slides from each brain block, deparaffinated, rehydrated, and dried, and tissue was peeled off and transferred to 1.5‐mL tubes. After deparaffinization, tissue was incubated in the Extraction Buffer (EXB) Plus, supplemented with β‐mercaptoethanol, at 100°C for 20 min and then at 80°C for 2 h on a thermomixer, following the manufacturer's instructions. The tubes were then centrifuged for 15 min at 14,000 g at 4°C, and the supernatant (soluble fraction) was collected, aliquoted, and stored at −80°C until further processing.

Total protein concentration in the supernatant was measured using the detergent compatible (DC)li Assay Kit (Biorad, Hercules, CA, USA) with bovine albumin as standard. Lysate samples (10 µg protein/lane) were separated by SDS‐PAGE, transferred to PVDF membranes using the Trans‐Blot Turbo Transfer System (BioRad, Hercules, USA), and non‐specific sites blocked before incubating with the primary antibodies overnight at 4°C. After washing, blots were incubated with secondary peroxidase‐linked antibodies. Primary antibodies used were 4G8 mouse anti‐Aβ clone (1:1000) and rabbit anti‐β actin (1:1000) (ABIN 72430, Antibodies online, Aachen, Germany). After thorough washing, secondary HRP‐conjugated anti‐mouse (Promega, Madison, WI, USA) and anti‐rabbit (Cell Signaling Technology, Danvers, MA, USA) antibodies were applied. Protein detection and quantification were performed using Bio‐Rad Clarity Max ECL substrate, the Biorad ChemiDoc Imaging System, and Image LabTM Version 6.0.0 software (Bio‐Rad Laboratories).

Statistical differences between groups were assessed using one‐way ANOVA followed by a post hoc LSD test using GraphPad Prism, with *p* < 0.05 considered significant. Data are expressed as means ± SEM (*n* = 3) (Figure S2).

## 3 RESULTS

### 3.1 Total brain amyloid burden is decreased in AQP4 facilitator‐treated animals and increased in AQP4 inhibitor‐treated APPPS1 animals

**FIGURE 1:**
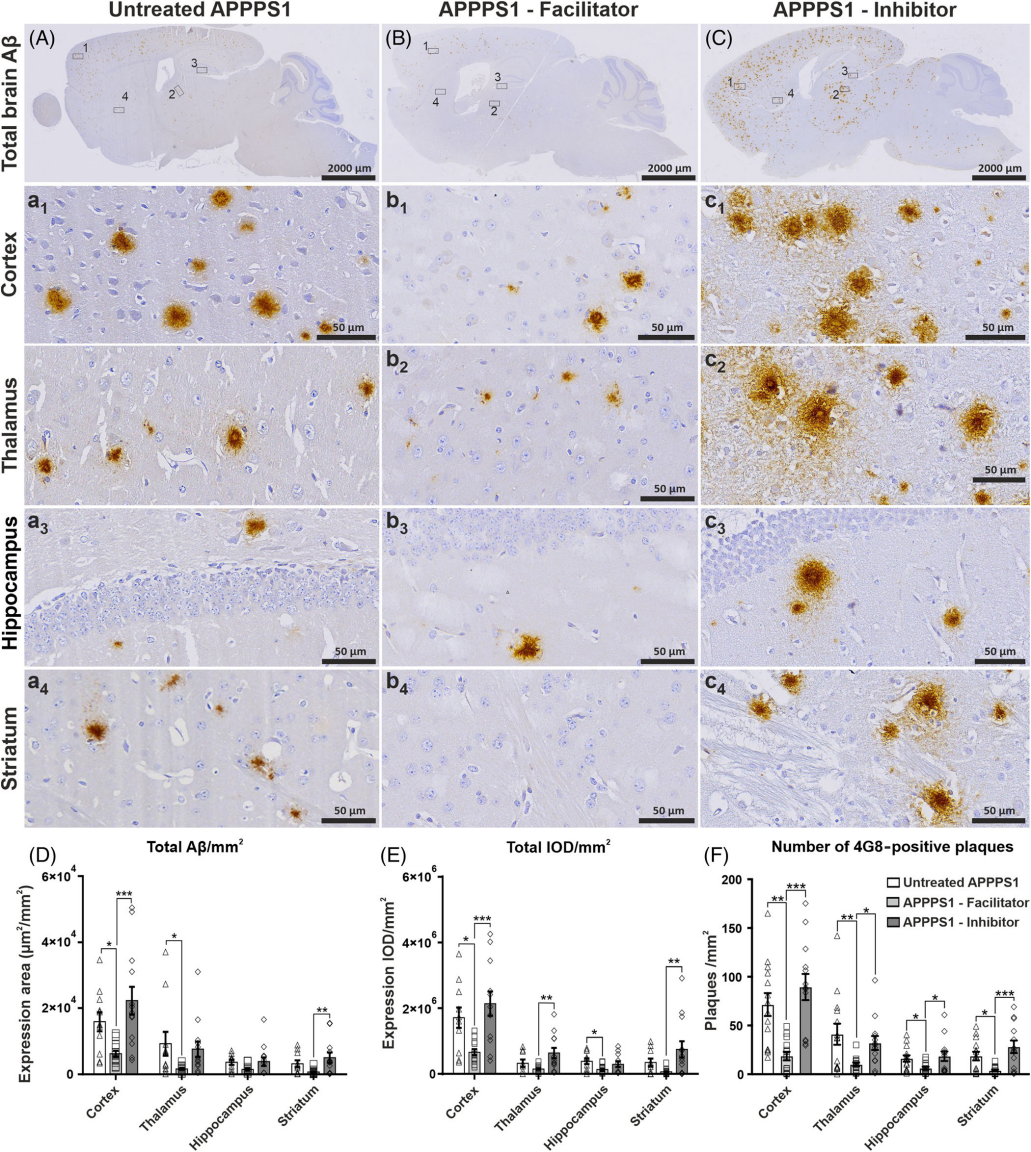
Total amyloid burden studied on unbiased whole‐brain scans after anti‐Aβ immunohistochemistry. Brains of 3‐month‐old untreated APPPS1 mice show moderate amyloid depositions throughout the brain (A), including cortex (a1), thalamus (a2), hippocampus (a3), and striatum (a4), while facilitator‐treated animals show a considerably lower amyloid burden (B, b1–b4), and inhibitor‐treated animals show considerably higher amyloid burden in all these regions (C, c1–c4). Image analysis on whole‐brain scans showed that facilitator‐treated animals had the smallest Aβ burden (D), IOD (E), and average total number of distinct plaques (F), while the inhibitor‐treated mice tended to exhibit the highest values on these parameters. Significance is shown for one‐way ANOVA followed by a post hoc Fisher's least significant difference test (*p < 0.05; **p < 0.01; ***p < 0.001). Data are expressed as means ± SEM. APPPS1, amyloid precursor protein presenilin 1; IOD, integrated optical density; SEM, standard error of the mean.

We first assessed the influence of AQP4 inhibition and facilitation on the total Aβ brain burden compared to untreated animal APPPS1 model. Slides immunostained for 4G8 antibody revealed that the AQP4 inhibitor‐treated animals exhibited the overall highest level of amyloid plaques, while the AQP4 facilitator‐treated group showed the smallest amounts of deposits on whole‐brain sagittal scans (Figure 1A–C). The differences were found in all the evaluated relevant anatomical regions, namely, the cortex (Figure 1Aa1–c1), thalamus (Figure 1Aa2–c2), hippocampus (Figure 1Aa3–c3), and striatum (Figure 1Aa4–c4). In the non‐transgenic WT group, 4G8 immunostaining showed no detectable signal (data not shown).

Moreover, the morphology of the plaques also seemed to be different after the treatments with predominantly dense‐diffuse and diffuse‐type plaques in the untreated animals (Figure 1Aa1–a4). Among the treatment groups, the facilitator‐treated animals seemed to deposit more compact plaques, with more visible dense cores and less diffuse coronas, especially in the cortex (Figure 1Bb1). On the other hand, the inhibitor‐treated group demonstrated mostly larger dense‐core plaques containing both large cores and abundant diffuse material around these central cores in not only cortex, but also thalamus, hippocampus, and striatum (Figure 1Cc1–c4).

We further explored the density of the plaques as 4G8 expression areas, IOD, and number of individual plaques, averaged per area unit. Thus, direct quantification of the total immunostained area by anti‐Aβ antibody 4G8 showed the TGN‐073 facilitator induced a massive drop of the total Aβ burden in all anatomical regions analyzed (Figure 1D). For the cortex, which showed the highest plaque areas (6135 ± 891.9 µm^2^/mm^2^), the difference was statistically significant compared to both control (15,861 ± 2902.3 µm^2^/mm^2^, *p* = 0.031) and inhibitor‐treated animals (22,286 ± 4189.0 µm^2^/mm^2^, *p* < 0.001). On the other hand, for the cortex, the inhibitor‐treated mice tended to exhibit the highest plaque burden, although the difference attained statistical significance only when compared to facilitator‐treated animals. Moreover, the TGN‐073 treatment induced a uniform decrease in plaque loads with minimal interanimal variability compared to both untreated and inhibitor‐treated animal groups.

Considering the total immunostained IODs, all studied brain regions clearly showed less compact and less dense plaques for the facilitator‐treated animals, and for the cortex this decrease (659,529 ± 93,509.3) was statistically significant when compared to both control (1,711,885 ± 310,647.4, *p* = 0.011) and inhibitor‐treated animals (2,136,499 ± 369,177.6, *p* < 0.001) (Figure 1E). The decrease compared to the inhibitor was also persistent for thalamus (635,390 ± 159,727.5 vs 148,805 ± 28,917.3, *p* = 0.003) and striatum (747,604 ± 247,009.7 vs 62,030 ± 25,059.8, *p* = 0.005). The inhibitor seemed to exhibit the highest signal density for all anatomical areas except hippocampus, probably due to the relatively low number of plaques here.

We next analyzed the average total number of plaques for each of the three treatment groups in the four anatomical areas as identified in whole scanned slides (Figure 1F). The facilitator revealed a notable decrease in total plaque number for all areas, and this difference was statistically significant compared to both untreated and inhibitor‐treated animal groups (*p* at least < 0.05). The inhibitor‐treated mice tended to exhibit the highest number of plaques; however, the difference was not statistically significant compared to the control group for any of the four regions.

### 3.2 Frequencies of vessel‐associated plaques decrease with AQP4 facilitation

**FIGURE 2:**
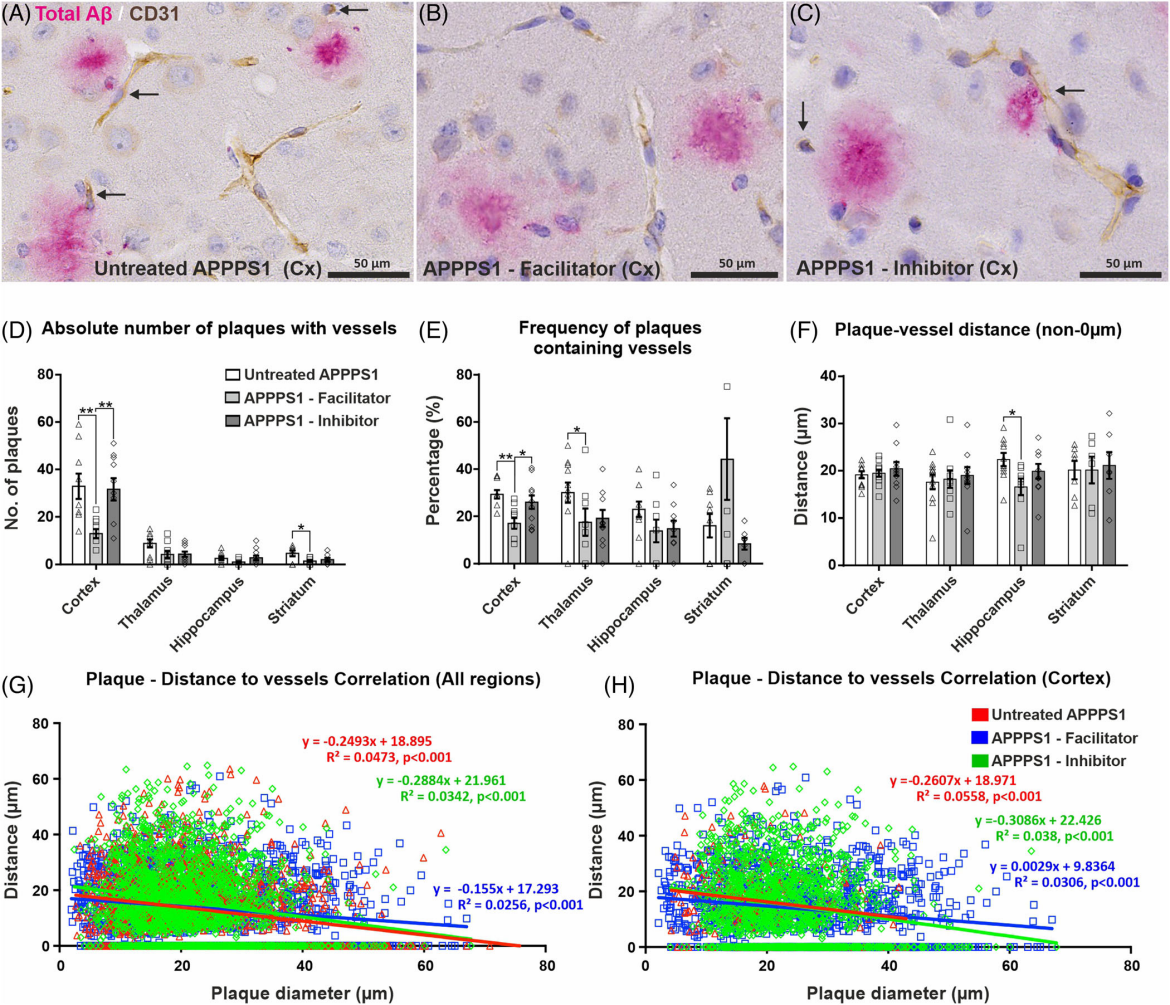
Analysis of relationship between blood vessels and amyloid plaques. Representative examples of cortical images co‐stained for total Aβ (4G8, red) and blood vessels (CD31, brown) demonstrate that blood vessels are proximal to or in direct contact (arrows) with amyloid plaques in all three animal groups (A–C). Plaque–vessel association was analyzed as absolute number of plaques in contact with at least one vessel (D), as frequencies of plaques in contact with at least one vessel (E), and as the average plaque–vessel distances (without considering here the plaques at 0 µm distance) (F). Finally, the plaque–vessel distances were also correlated with diameter of the plaques (data shown for all regions) (G) and cortices (H), with the lowest correlation slopes being recorded for facilitator‐treated animals. For panles, D–F, significance is shown for one‐way ANOVA followed by a post hoc Fisher's least significant difference test (*p < 0.05; **p < 0.01; ***p < 0.001). Data are expressed as means ± SEM. Aβ, amyloid beta; SEM, standard error of the mean.

Considering the proven mechanisms of perivascular amyloid drainage, vasculotropic plaque formation, and our data on altered sizes of dense‐core plaques and their corona, we next evaluated the average number of plaques containing a blood vessel or in direct contact with blood vessels, by assessing double stained slides for CD31 (blood vessel marker) and 4G8 (Figure 2A–C).

Plaque–vessel association analysis revealed that the facilitator‐treated animals had the lowest number of plaques with vessels among all areas, and for the cortex the differences were also statistically significant compared to both control (13.0 ± 1.91 vs 32.8 ± 5.32, *p* = 0.002) and inhibitor‐treated groups (31.6 ± 4.67, *p* = 0.003) (Figure 2D). A significant difference was obtained also for the striatum of facilitator‐treated animals compared to the control group (1.4 ± 0.48 vs 4.7 ± 1.27, *p* = 0.035). On the other hand, control animals showed a tendency toward the highest number of plaques being associated with a blood vessel among the three studied groups.

Analyzing the percentages of plaques containing a blood vessel inside or in direct contact with the vessel, versus plaques not associated with vessels, the facilitator group showed a notable drop in the plaque–vessel association compared to control and inhibitor‐treated animals for cortex and hippocampus (Figure 2E). For the cortices, due to the higher number of deposits, the variations were lower than for the other regions, and the facilitator‐treated animals (17.8% ± 1.73%) showed significantly lower plaque–vessel associations compared to both untreated (27.9% ± 2.26%, *p* = 0.003) and inhibitor‐treated animals (25.6% ± 2.53%, *p* = 0.014). Although the inhibitor‐treated mice had a decreasing trend in the association frequency compared to control animals, this did not reach statistical significance (*p* = 0.464).

We next explored the distances from dense or dense‐diffuse amyloid plaques (defined as diffuse neuropil deposits but with clearly demarcated borders) to the nearest blood vessel identifiable on double immunohistochemistry for total Aβ and CD31. Vessels touching the Aβ plaques or contained within plaques were counted as having a 0 µm distance to the respective plaque. Although there were subtle variations, with the facilitator‐treated animals exhibiting a tendency toward smaller plaque–vessel distances compared to inhibitor‐treated animals, these differences did not attain statistical significance (data not shown).

Disregarding the plaques that were in direct contact with vessels, our data showed a tendency for inhibitor‐treated animals to develop plaques slighter farther away from blood vessel endothelia compared to facilitator‐treated and control animals; however, the differences did not attain statistical significance (*p* > 0.05) (Figure 2F). The facilitator group did not differ significantly from the control group, except for the hippocampus, where there was a significant decrease in distance for the facilitator‐treated animals (16.6 ± 1.75 µm) compared to the control animals (22.3 ± 1.41 µm, *p* = 0.015).

Next, we studied whether the diameter of the plaques was proportional to the distance to the nearest blood vessel on double‐immunohistochemically stained slides (Figure 2G,H). Overall, for all anatomical regions pooled together, there was a small indirect correlation of the plaques’ diameter with the distances to the neighboring blood vessels for all groups (*r* = −0.217, control; *r* = −0.160, facilitator; and respectively *r* = −0.184, inhibitor, *p* < 0.0001) (Figure 2G). The lowest correlation degree was thus recorded for facilitator‐treated animals, which also showed a statistically significant difference when comparing these with control animals’ correlation coefficient (Fisher's *Z* = −1.7137, *p* = 0.0433), but not with the inhibitor‐treated animals’ correlation coefficient (Fisher's *Z* = −0.7760, *p* = 0.2189).

We also replicated this analysis only for the cortices, as this region harbors by far the highest density of Aβ depositions (Figure 2H). Here, the diameter–distance indirect correlations were slightly stronger compared to the overall all‐areas assessments (*r* = −0.236, control; *r* = −0.174, facilitator; and, respectively, *r* = −0.194, inhibitor, *p* < 0.0001). The correlation degree decreased mostly for facilitator‐treated animals, with a statistically significant difference with the control animals’ correlation coefficient (Fisher's *Z* = −1.7311, *p* = 0.0417), but again, not with the inhibitor‐treated animals’ correlation coefficient (Fisher's *Z* = −0.6091, *p* = 0.2712). Remarkably, there was also no significant difference between correlation coefficients for control and inhibitor‐treated animals (Fisher's *Z* = 1.1419, *p* = 0.1267).

### 3.3 Morphology of amyloid plaques is also influenced by AQP4 modulation

**FIGURE 3:**
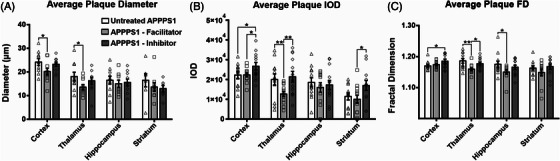
Morphological analysis of amyloid plaques (as evaluated after 4G8 immunohistochemistry) assessed maximum diameters, signal IOD, and FD. Diameter analysis showed that facilitator‐treated animals had a tendency for the smallest deposits among the three animal groups (A), the average IOD revealed that the densest deposits occurred for the inhibitor‐treated animals (B), and the FD showed that inhibitor‐treated group also seemed to harbor mostly dense‐core types of deposits with high FD values (C). Significance is shown for one‐way ANOVA followed by a post hoc Fisher LSD test (*p < 0.05; **p < 0.01; ***p < 0.001). Data are expressed as means ± SEM. FD, fractal dimension; IOD, integrated optical density; SEM, standard error of the mean.

We investigated whether the average plaque diameters were altered after the two types of treatment (Figure 3A). The facilitator showed a strong tendency toward depositing smaller plaques when compared to control and inhibitor‐treated animals for cortex, thalamus, and hippocampus, regions with the highest number of plaques. For cortex and thalamus, the facilitator showed significantly smaller plaque diameters (20.2 ± 1.35 µm; 13.5 ± 1.10 µm) compared to control animals (24.2 ± 1.46 µm, *p* = 0.05; 18.1 ± 1.82 µm, *p* = 0.044), while the inhibitor exhibited intermediate values without statistically significant differences (23.3 ± 0.93 µm, *p* = 0.096; 16.3 ± 1.51 µm, *p* = 0.213).

We next assessed the average plaque signal density IOD (Figure 3B), and here the facilitator‐treated animals showed values very close to the control groups for the cortex (22,292 ± 976.1 vs 23,181 ± 1807.1, *p* > 0.05), hippocampus (18,206 ± 2984.1 vs 18,554 ± 2442.6, *p* > 0.05), and striatum (100,007 ± 1980.8 vs 11,429 ± 1813.0, *p* > 0.05), while the inhibitor exhibited the most compact plaques, with the highest IOD values (26,749 ± 1906.7, *p* = 0.05 for cortex; 21393 ± 2694.8 vs 12681 ± 1280.3, *p* = 0.009 for thalamus; and, respectively, 16,992 ± 2522.6, *p* = 0.024 for striatum). The fact that the total IOD for facilitator‐treated animals showed significantly lower values compared to the control group (Figure 1E) while averaged plaque IOD showed similar values for facilitator and control groups (Figure 3B) suggests that average individual plaque densities were remarkably similar, but with an important decrease in the total Aβ burden for facilitator‐treated animals, a fact that has been clearly demonstrated when assessing total non‐N truncated Aβ burden expression areas (Figure 1D). On the other hand, the total Aβ burden patterns taken together with the total and average plaque IOD analysis revealed that the inhibitor‐treated animals exhibited the largest Aβ depositions, as well as the densest individual plaques, as detected by immunohistochemistry, compared to both control and facilitator groups (Figure 3B).

To assess the complexity of compact versus diffuse patterns of amyloid deposition within plaques, we evaluated the FD as the mathematical denominator of complexity of the immunohistochemistry patterns of expression (Figure 3C). For all anatomical regions except cortex, the facilitator showed a clear tendency toward producing plaques with the lowest FD values, while the inhibitor group consistently showed higher FD values compared to the facilitator‐treated animals. For thalamus and hippocampus, the FDs were significantly lower for the facilitator group compared to control animals (1.16 ± 0.004 vs 1.19 ± 0.007, *p* = 0.002 for thalamus and 1.15 ± 0.006 vs 1.17 ± 0.010, *p* = 0.042 for hippocampus), while there were no statistically significant differences for cortex and striatum areas.

Altogether, morphological assessments corroborating area, diameter, IOD, and FD analyses suggest that since AQP4 facilitator and inhibitor action is based on the modulation of the perivascular drainage of the ISF, which takes up mostly soluble Aβ isoforms, this modulation alters the Aβ40/Aβ42 ratio of the remaining interstitial peptides, with drastic changes in amyloid diffusivity and clearance by mechanisms that are evolved to cope with a “physiological” proportion of these isoforms. Thus, it can be conceived that, on the one hand, facilitating drainage will leave behind diffuse and uncompacted fast‐precipitating Aβ42‐rich material, while, on the other hand, blocking drainage will lead to an increase in total Aβ burden, resulting in more numerous and larger dense‐core types of plaques, with more inner and edge irregularities.

### 3.4 Increased Aβ42 ratio in both AQP4 inhibitor‐ and facilitator‐treated APPPS1 animals

**FIGURE 4:**
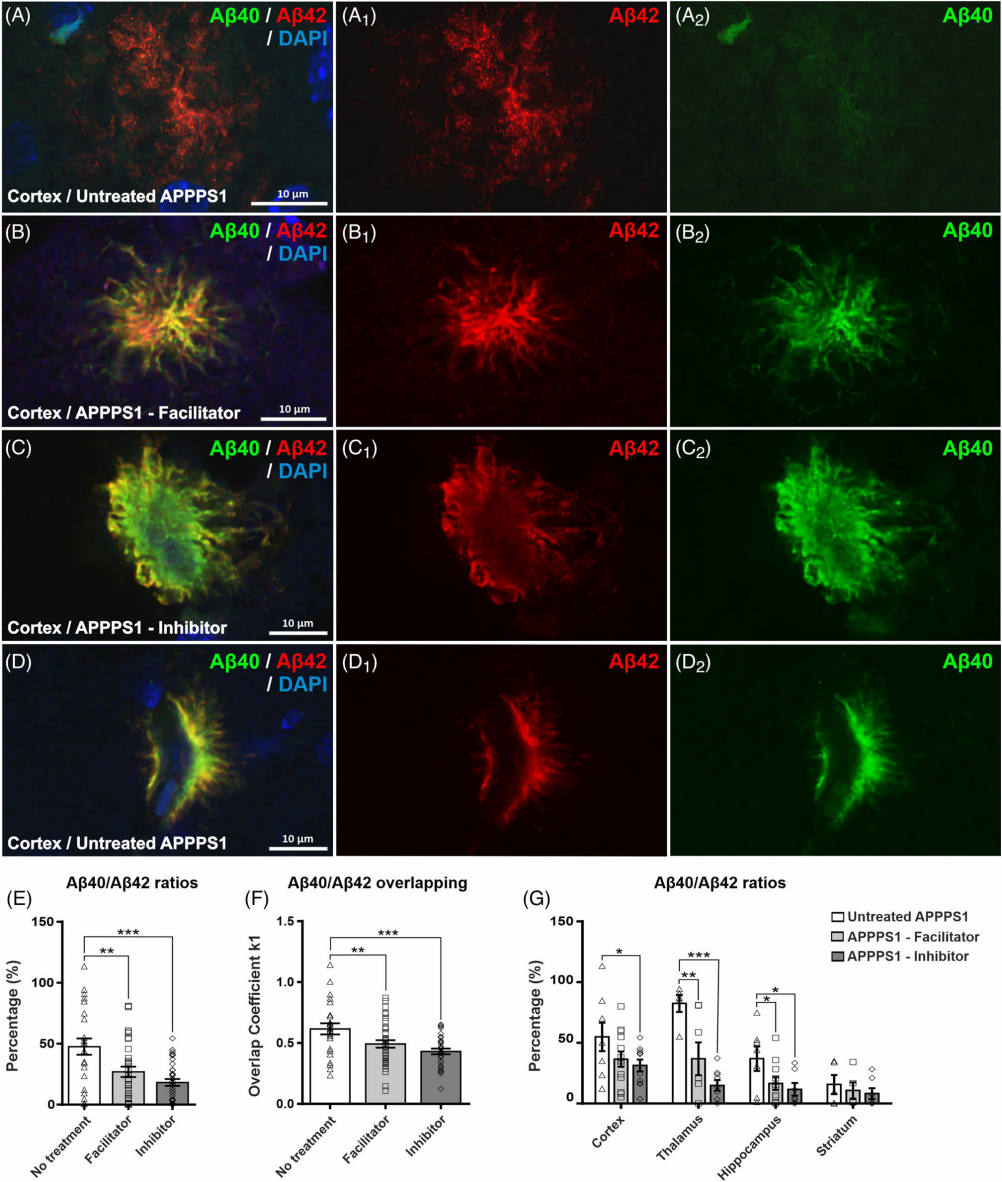
Types of amyloid deposits found to different extents and sizes in all three APPPS1 animal groups, and analysis of Aβ42 and Aβ40 content. Double immunohistochemistry for Aβ40 (green) and Aβ42 (red) imaged in confocal microscopy reveal plaques ranging from typically diffuse (A, A1, A2), to dense (B, B1, B2), dense‐core (C, C1, C2), and dysphoric angiopathy (D, D1, D2). While the diffuse plaques and the diffuse per‐core material of the dense deposits contain mostly Aβ42, the more compact the plaque becomes, the more Aβ40 it will contain. Aβ42 was the predominant isoform in all animals, and averaging for all plaques in all areas revealed that inhibitor‐treated animals exhibited the highest relative increase in Aβ42 compared to untreated animals, with the facilitator‐treated groups showing intermediate values (E), and this increase in relative Aβ42 is also linked to a decrease of the overlapping coefficients between the two isoforms (F). Segregating the Aβ40/Aβ42 ratio, the same pattern is maintained for all anatomical regions (G). Significance is shown for one‐way ANOVA followed by a post hoc Fisher's least significant difference test (*p < 0.05; **p < 0.01; ***p < 0.001). Data are expressed as means ± SEM. Aβ, amyloid beta; APPPS1, amyloid precursor protein presenilin 1; SEM, standard error of the mean.

Next, we assessed the co‐expression of Aβ42 and Aβ40 deposited fractions, as well as their colocalization degrees in our animal groups, after AQP4 inhibition and or facilitation treatments. On more than 600 amyloid plaques analyzed individually, with varying morphologies from diffuse to dense‐diffuse and dense‐core plaques, as well as dysphoric angiopathy, Aβ42 consistently predominated over the Aβ40 in all animal groups (Figure 4A–D).

Analysis of the immunolabeled areas of Aβ42 and Aβ40 fractions revealed a highly significant increase in Aβ42 deposited in treated animals relative to Aβ40 (i.e., decreased Aβ40/Aβ42 ratio), for both the facilitator‐treated (26.9% ± 4.21%, *p* = 0.002) and inhibitor‐treated groups (18.2% ± 2.80%, *p* < 0.0001) compared to control animals (47.6% ± 6.67%), without any significant difference between the treated groups (*p* = 0.151) (Figure 4E). Taken together with a decrease of the total deposited Aβ burden for facilitator‐treated animals (Figure 1D,E), the decreased Aβ40/Aβ42 ratio for this group suggests an absolute decrease in both isoforms, but especially for the Aβ40, which fits well with the knowledge that activating the AQP4 ISF drainage mostly clears soluble Aβ fractions. On the other hand, a further decrease in the Aβ40/Aβ42 ratio for the inhibitor‐treated animals (Figure 4E), corroborated here by a solid increase in the total Aβ burden (Figure 1D,E), translates into a massive accumulation of both isoforms, but mostly Aβ42 in these animals, conceivably due to the increased total Aβ fractions that result from blocking the AQP4 perivascular drainage pathways.

The same pattern was present when we studied the overlapping ratio between Aβ40 and Aβ42, as expressed by double immunofluorescence, again with a highly significant decrease for both facilitator‐treated (0.49 ± 0.03, *p* = 0.01) and inhibitor‐treated (0.43% ± 0.02%, *p* < 0.0001) groups versus control animals (0.62% ± 0.005%). No significant difference was observed between the two treatment groups (*p* = 0.162) (Figure 4F).

Lastly, performing similar Aβ40/Aβ42 ratio analysis separately for the four anatomical regions, we observed the same pattern for all areas (Figure 4G), although significant drops for facilitator and inhibitor animals were maintained only for thalamus (control [82.5% ± 7.07%] vs facilitator [36.9% ± 13.60%, *p* = 0.004], respectively, vs inhibitor [14.9% ± 4.31%, *p* < 0.0001]) and hippocampus (control [37.0% ± 10.11%] vs facilitator [16.5% ± 5.19%, *p* = 0.041], respectively, vs inhibitor [11.7% ± 5.20%; *p* = 0.024]), while for the cortex, the differences attained statistical significance only for the inhibitor group (31.4% ± 4.81%) versus control animals (54.9% ± 11.84%, *p* = 0.044).

### 3.5 Fibrillar amyloid deposits decrease after AQP4 facilitation

**FIGURE 5:**
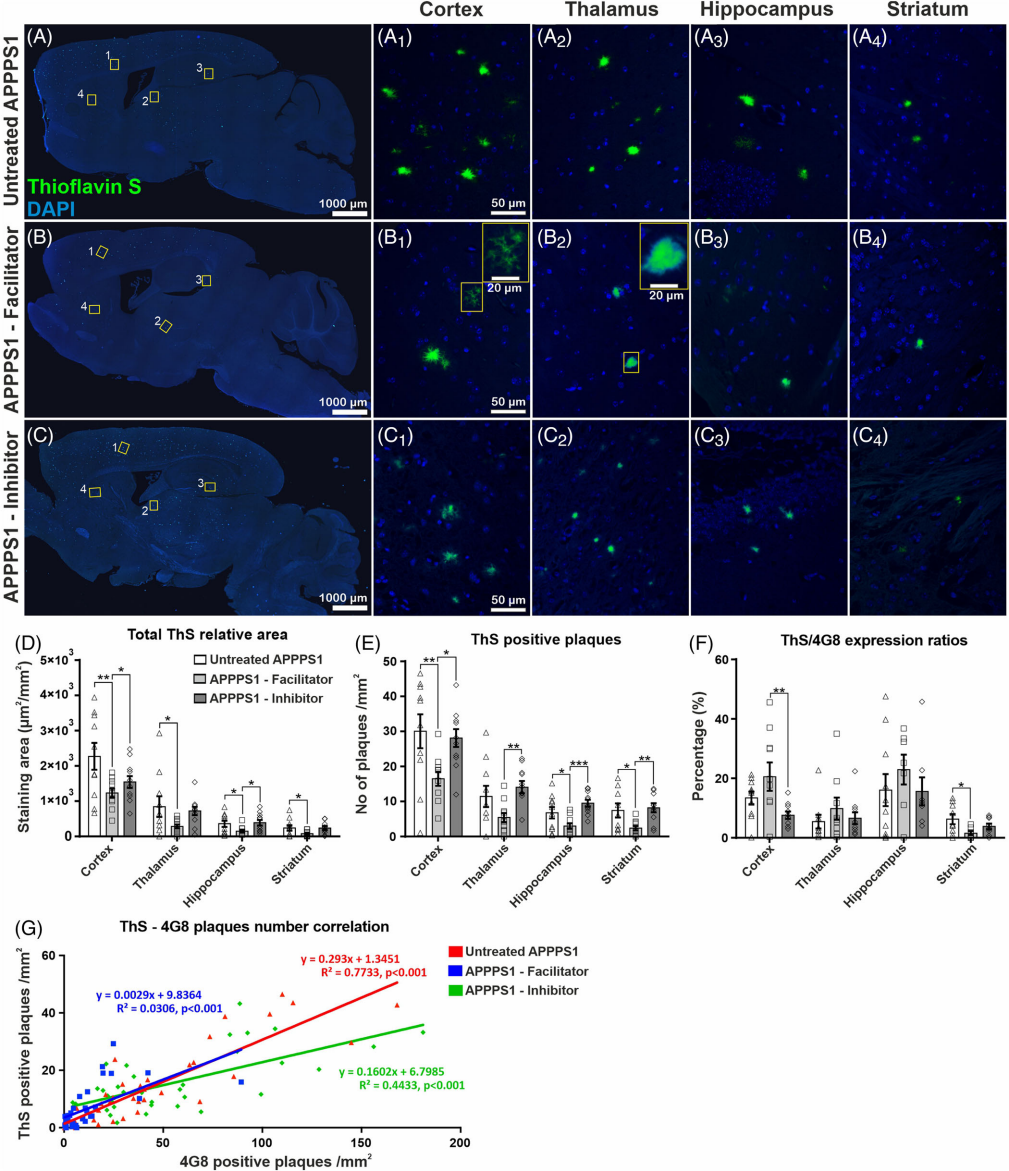
Fibrillar amyloid deposits assessed via ThS staining. Fully scanned brain sections stained with ThS reveal the extent of fibrillar amyloid deposits in the three APPPS1 animal groups (A–C), with representative deposits exhibiting patterns of focal fibril meshes and compact dense cores (enlarged insets in b1 and b2). ThS‐positive plaques were less abundant in AQP4 facilitator‐treated animals compared to untreated and inhibitor‐treated APPPS1 mice, both in terms of staining areas (D) and plaque counts (E). Analysis of ThS‐positive plaques relative to total plaques identified by immunohistochemistry showed a trend toward more compact amyloid deposits in facilitator‐treated mice (F). A strong correlation between the number of ThS‐positive plaques and 4G8 immunohistochemistry‐positive plaques was observed in untreated APPPS1 plaques, which decreased in both treatment groups (G). Significance was determined by one‐way ANOVA followed by a post hoc Fisher's least significance difference test (*p < 0.05; **p < 0.01; ***p < 0.001). Data are expressed as means ± SEM. Aβ, amyloid beta; APPPS1, amyloid precursor protein presenilin 1; AQP, aquaporin; SEM, standard error of the mean; ThS, thioflavin S.

AD is characterized by a variety of Aβ plaques, from diffuse to highly compact ones, with dense cores that stain with fibril‐binding dyes such as ThS. To assess the impact of treatments on fibrillar Aβ, we analyzed ThS‐stained sections from three pathology groups (Figure 5A–C). ThS‐positive plaques varied from focal fibril meshes to compact dense cores of variable sizes across brain regions (Figure 5Bb1,b2).

Quantification of the relative ThS staining area showed that AQP4 facilitation led to a significant reduction in fibrillar deposits in all brain regions compared to untreated animals (Figure 5D). In contrast, the AQP4 inhibitor also showed a decrease in fibrillar plaque load, particularly in the cortex, but this change was not statistically significant. In the cortex and hippocampus, the AQP4‐facilitator group exhibited the lowest ThS‐stained areas compared to both control and inhibitor‐treated groups (cortex: 1229.5 ± 121.69 µm^2^/mm^2^ vs 2270.3 ± 381.06 µm^2^/mm^2^, *p* = 0.005, and vs 1544.3 ± 137.67 µm^2^/mm^2^, *p* = 0.043; hippocampus: 149.5 ± 37.88 µm^2^/mm^2^ vs 360.1 ± 93.49 µm^2^/mm^2^, *p* = 0.005, and vs 388.5 ± 78.23 µm^2^/mm^2^, *p* = 0.025).

The number of ThS‐positive plaques was also significantly reduced in the facilitator‐treated group, with the inhibitor showing similar or slightly higher plaque numbers than those of the control group (Figure 5E). Notably, except for the thalamus, facilitator‐treated animals exhibited the lowest number of ThS‐positive plaques compared to both untreated and inhibitor‐treated groups: cortex: (16.4 ± 2.00/mm^2^ vs 30.0 ± 4.84/mm^2^, *p* = 0.007, and vs 28.1 ± 2.53/mm^2^, *p* = 0.016), hippocampus (2.9 ± 0.73/mm^2^ vs 6.7 ± 1.60/mm^2^, *p* = 0.025, and vs 9.5 ± 0.95/mm^2^, *p* < 0.0001), and striatum (2.4 ± 0.64/mm^2^ vs 7.4 ± 1.88/mm^2^, *p* = 0.014, and vs 8.2 ± 1.26/mm^2^, *p* = 0.004 for striatum).

When examining the relative percentage of ThS‐positive staining compared to 4G8 immunoexpression (Figure 5F), the facilitator‐treated group exhibited a higher ThS/4G8 ratio in most regions except the striatum. In the cortex, the facilitator group had a significantly higher ratio compared to the inhibitor group (20.5% ± 5.34% vs 7.5% ± 1.28%, *p* = 0.006) but not compared to control (13.4% ± 2.21%, *p* = 0.116). The striatum showed the smallest ThS/4G8 ratio in the facilitator group (1.5% ± 0.68% vs controls at 6.2% ± 1.68%, *p* = 0.015). Although absolute ThS and 4G8 staining areas were significantly reduced for facilitator‐treated animals, the remaining amyloid deposits showed, thus, more fibrillar aggregation. This correlated with perivascular Aβ clearing, especially for Aβ40 (Figure 4G), and suggests that the increased Aβ42 peptide is more prone to rapid fibrillization and aberrant oligomerization.

We also explored the correlation between ThS‐positive plaques and the total plaques identified by immunohistochemistry (Figure 5G). For the control group, there was a strong correlation between the two types of plaques (*r* = 0.879, *p* < 0.0001), which decreased for both the facilitator (*r* = 0.614, *p* < 0.0001) and inhibitor groups (*r* = 0.665, *p* < 0.0001). The correlation coefficients were significantly lower for both treatment groups compared to controls (Fisher's *Z* = 2.7489, *p* = 0.006; Fisher's *Z* = −2.3794, *p* = 0.0173), but there was no significant difference between the facilitator and inhibitor groups (Fisher's *Z* = 0.3594, *p* = 0.7193). This loss of correlation reflects the opposing effects of the facilitator and inhibitor treatments on plaque numbers, Aβ40/Aβ42 ratios, ThS content, and ThS/4G8 ratios, suggesting a disruption in the relationship between the fibrillar and total deposited amyloid.

### 3.6 Soluble Aβ content decreases after AQP4 facilitation and increases after AQP4 inhibition

**FIGURE 6:**
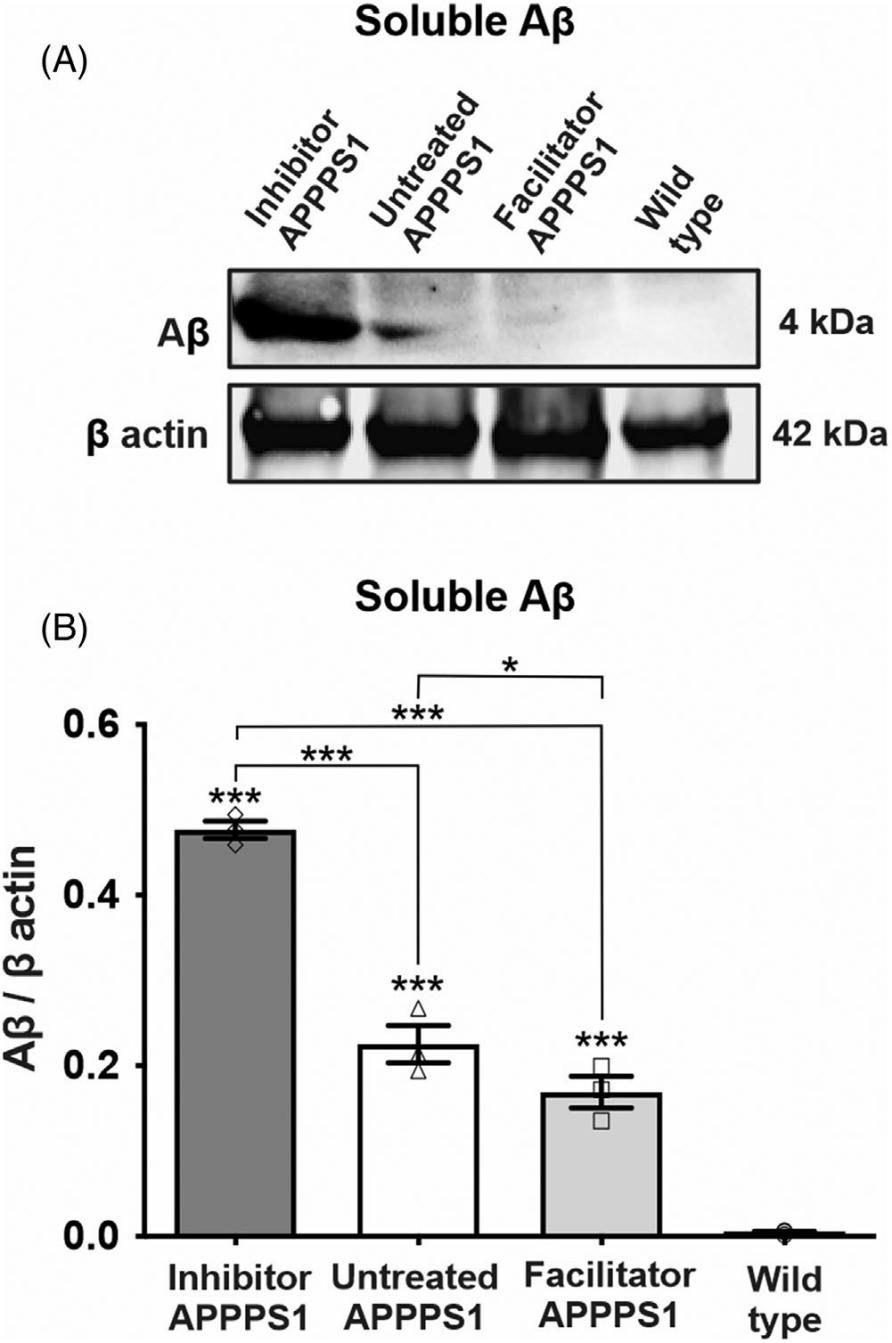
AQP4 inhibition and facilitation alter soluble Aβ peptide levels. Western blot analysis of the levels of soluble amyloid β peptide (Aβ) levels for each experimental group is shown, with β actin used as loading control (A). Normalized Aβ values showed significant differences between groups, with the AQP4 inhibitor‐treated animals showing significantly higher levels and the AQP4 and the facilitator‐treated animals exhibiting significantly lower levels compared to untreated animals. (B) Significance was determined by one‐way ANOVA followed by a post hoc Fisher's least significance difference test (*p < 0.05; ***p < 0.001). Data are expressed as means ± SEM. Aβ, amyloid beta; AQP, aquaporin; SEM, standard error of the mean.

Western blot analysis of the soluble Aβ fraction revealed a marked increase in soluble Aβ levels in the AQP4 inhibitor‐treated animals (0.47 ± 0.010) compared to both the facilitator‐treated (0.16 ± 0.097, *p* < 0.001) and untreated PSAPP1 mice (0.22 ± 0.130, *p* < 0.001) (Figure 6). While the AQP4 inhibitor treatment increased soluble Aβ levels, AQP4 facilitation also led to a notable reduction in soluble Aβ compared to the untreated APPPS1 mice (*p* = 0.031).

### 3.7 Improved behavioral features for facilitator‐treated animals

**FIGURE 7:**
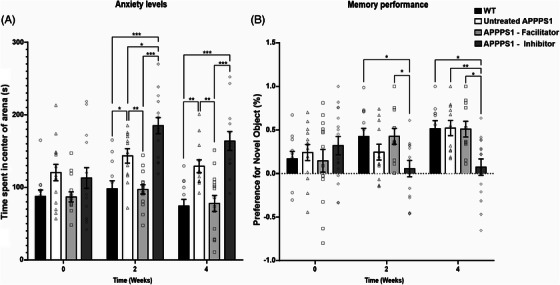
Behavior changes during treatments. Baseline testing of anxiety revealed no difference between the tested animals. Starting with the second week of treatments, APPPS1 animals treated with TGN‐020 showed an increase in average time spent in the open, compared to the time spent by WT mice and that of control APPPS1 animals. The behavior was further amplified after 4 weeks of treatment. While a lack of anxiety seems to be observed in APPPS1 controls and TGN‐020‐treated animals, TGN‐073‐treated animals display similar levels of anxiety compared to WT animals (A). Baseline testing of memory revealed no difference between the tested animals. Starting with the second week of treatment, animals receiving daily injections of TGN‐020 fail to identify novel objects, displaying no preference for the new object, compared to APPPS1 controls and WT animals. APPPS1 animals that received TGN‐073 did not display any loss in memory compared to the WT group. The administration of TGN‐020 for 4 weeks rendered the TGN‐020 group unable to distinguish between the new and familiar object (B). Significant interactions are illustrated based on a REML estimation analysis; *p < 0.05; **p < 0.01; ***p < 0.001. Data are expressed as means ± SEM. APPPS1, amyloid precursor protein presenilin 1; SEM, standard error of the mean.

At the start of the experiments, all tested animals (aged 2 months) showed similar levels of anxiety and short‐term memory, as tested using the OF and NOR techniques (Figure 7A,B). Statistical analysis was performed using the mixed‐effects model (restricted maximum likelihood [REML]) to evaluate the behavior of the APPPS1 animals. The model investigated the raw factor (time), referred to as Sessions, the column factor (different treatments), referred to as Treatment, and the interaction between the two (Interaction). REML analysis of anxiety levels of APPPS1 mice revealed significant Interaction (F[6,99] = 2.837, *p* = 0.0136) between Sessions (F[1.9, 94.35] = 8.007, *p* = 0.0007) and Treatment (F[3, 54] = 28.79, *p* < 0.0001) (Figure 7A). Post hoc analysis using Tukey's multiple comparisons test revealed that anxiety decreased in untreated APPPS1 mice and APPPS1 animals treated with TGN‐020, as shown by the increased time spent in open space from 120.4 ± 43.63 s and 112.9 ± 56.89 s to 143.4 ± 34.56 s, respectively, 185.0 ± 42.71 s, compared to the average 98.1 ± 38.39 in WT mice (*p* = 0.0008 and *p* < 0.0001), with no change in anxiety being seen in WT and animals treated with TGN‐073, regardless of session (Figure 7A).

While a lack of anxiety seems to be observed in APPPS1 animals in all sessions, animals treated with TGN‐020 were significantly less anxious starting from week 2 of treatment. Treating APPPS1 animals with TGN‐073 seemed to restore this behavior, as post hoc analysis did not reveal any differences between the two, both at 2 weeks (98.1 ± 38.39 s compared to 97.2 ± 24.81 s, *p* > 0.05) and 4 weeks (74.4 ± 31.91 s compared to 77.9 ± 42.65 s, *p* > 0.05) of treatment (Figure 7A).

REML analysis of APPPS1 mice memory revealed strong Interactions (F[6, 139] = 3.069, *p* = 0.0075) between Treatment (F[3, 139] = 3.675, *p* = 0.0138) and Sessions (F[1.9, 134.2] = 3.471, *p* = 0.0355) (Figure 7B). Post hoc analysis revealed that while the TGN‐020 treatment animals failed to identify novel objects starting at 2 weeks of treatment scoring 0.05 ± 0.36 compared to 0.24 ± 0.31 in APPPS1 controls (*p* = 0.4780) and 0.42 ± 0.32 in WT animals (*p* = 0.044), animals treated with TGN‐073 displayed no loss in memory (0.42 ± 0.30) compared to WT (*p* = 0.999) (Figure 7B). After 4 weeks of treatment, the differences between TGN‐020‐ and TGN‐073‐treated animals increased, with TGN‐020 animals being able to only have an average preference index of 0.07 ± 0.36 compared to 0.51 ± 0.29 in TGN‐073‐treated APPPS1 animals (*p* = 0.0132) (Figure 7B).

## 4 DISCUSSION

Based on the bidirectional flow of AQP4 in astrocyte endfeet, ISF containing solutes/catabolites drains outside the brain and diffuses along the basement membranes (BMs) of the capillaries and the BMs within tunica media of arterioles, and is finally collected by the meninges and lymphatics of the neck, a pathway known as the IPAD pathway.^18^, ^22^, ^46^, ^47^

In AD, despite the fact that age/genetic factors heavily modulate Aβ overproduction and alter the Aβ40/Aβ42 ratio, facilitating fibril formation,^48^, ^49^ the final accumulation of amyloid is a result of an imbalance between production and clearance mechanisms.^50^, ^51^ Studies on human pathology have shown that Aβ overproduction and age‐related vascular changes all lead to a failure of perivascular drainage, where accumulating Aβ initially in the vascular BM progresses to replace all of the vessel walls as CAA.^52^, ^53^ All these data support the vasculotropic plaque formation hypothesis, wherein insufficient drainage along the IPAD pathway leads to Aβ being deposited first on the abluminal side of the vascular BM, then replacing the complete wall as CAA, or even expanding in the neuropil as dysphoric angiopathy or neuritic plaques.^16^, ^17^

Utilizing in vivo two‐photon microscopy, we previously showed that TGN‐020‐mediated AQP4 inhibition with a single massive 400 mg/kg dose alters IPAD, leading to perivascular accumulation of Aβ40.^31^ Here, we further investigated the effects of AQP4 modulation on brain Aβ by utilizing 2‐month‐old APPPS1 mice, at the age when amyloid deposition begins.^36^ We showed that animals that received a 200 mg/kg daily i.p. dose of TGN‐020 inhibitor had a higher total Aβ burden and a larger number of plaques, which were also more compact compared to untreated APPPS1 animals. A recent study also reported increased Aβ accumulation in APPPS1 mice treated daily with a 10 mg/kg i.p. dose of TGN‐020 for 28 days, along with higher escape latency in the Y maze and Morris water maze compared to untreated mice.^35^ Additionally, a recent preprint suggests that TGN‐020 may have complex, not fully understood interactions with adenosine receptors, which could influence glymphatic drainage in vivo.^54^ Regarding the choice of dosages in our experiments, to our knowledge, no studies have established the minimum effective concentrations of TGN‐020 and TGN‐073 across different models, as their precise effects under non‐physiological conditions are still being investigated. Our selection of the 200 mg/kg dosage for both compounds was based on prior literature and empirical evidence. In vitro experiments on *Xenopus laevis* oocytes demonstrated effective concentrations as low as 3 µM for the inhibitor^55^ and 10 µM for the facilitator.^34^ Furthermore, previous studies including our own have shown 100 to 400 mg/kg unique doses of TGN‐020 successfully modulate post‐stroke edema^29^ and perivascular drainage of Aβ40.^31^

**FIGURE 8:**
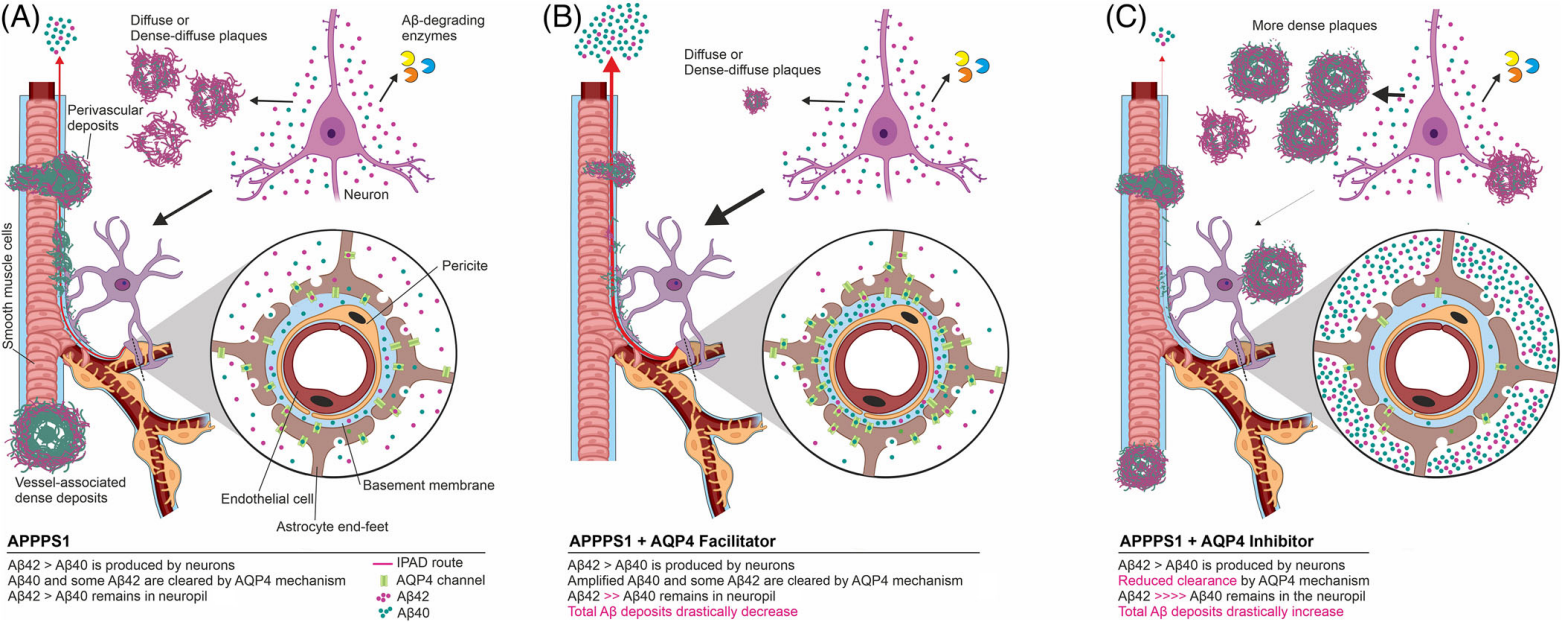
Schematic diagram of how AQP4 modulation influences clearing of Aβ from brain of APPPS1 mouse model. (A) In untreated animals, Aβ produced by neurons is (i) degraded in the neuropil by dedicated enzymatic mechanisms, (ii) deposited as diffuse or dense‐diffuse plaques not associated with blood vessels, and (iii) drained along IPAD pathways along laminar structures of vascular BM. At the level of the capillaries, the BM at the abluminal side of the endothelial cells split to enclose pericytes, but are almost universally bounded by astrocyte endfeet. Beginning with small arterioles, a complex laminar network of BM also surrounds the SMCs of tunica media, and around the astrocyte end feet now create the perivascular glia limitans. Diffusible Aβ species (readily soluble Aβ together with less soluble chaperone protein‐bound Aβ) are cleared by the astrocytes by endocytosis, which further cross into the subastrocytic BM matrix through AQP4 pores expressed on the abluminal surface of the astrocyte endfeet. Aβ further diffuses along till it reaches the BM surrounding SMCs. Insufficiency of the IPAD due to the overproduction of Aβ leads to its accumulation as small cusps of diffuse deposits of Aβ40/ Aβ42 along the perivascular astrocyte network or directly in the arterial tunica media. The initial nidi lead to further deposition of Aβ, leading to the complete collapse of (small) vascular branches, which are completely replaced by well‐formed dense and dense‐core plaques, with Aβ40 aggregating together with Aβ42 to form dense amyloid plaque cores. (B) In APPPS1 animals treated with the AQP4 facilitator, a gain of function of AQP4 ensures an augmented drainage of the parenchymal Aβ along the IPAD pathway. A preferential drainage of Aβ40 through AQP4 channels leads to decrease in both total Aβ as well as in the Aβ40/Aβ42 ratio. Plaques in the neuropil are formed, but due to the rapid clearance of Aβ through AQP4 channels, both the total number of parenchymal plaques along with their densities are significantly reduced compared to the untreated animal group. The rapid clearance of Aβ through the vascular route also does not allow precipitation of Aβ and plaque formation in association with blood vessels. (C) In APPPS1 animals treated with the AQP4 inhibitor, the IPAD clearance is mediated preferentially by alternative and less efficient mechanisms, such as the lipoprotein receptor‐related protein (LRP)‐ or AQP1‐mediated trans‐endothelial transport. Thus, the total Aβ is significantly increased in the neuropil. While excess absolute amounts of Aβ42 in the neuropil facilitates plaque seeding, the increased Aβ40 leads to faster growth, resulting in increased sizes and densities of dense and dense‐diffuse plaques compared to untreated and AQP‐facilitator treated animals. While this preferentially occurs around the perikarya, as neurons are the sole source of Aβ production in these mice, some vessel‐associated plaques are also observed due to impediments in Aβ clearance in IPAD pathways (artwork created with BioRender.com). Aβ, amyloid beta; APPPS1, amyloid precursor protein presenilin 1; AQP, aquaporin; BM, basement membrane; IPAD, intramural periarterial drainage; SMC, smooth muscle cell.

Conversely, we also showed here that APPPS1 animals receiving the TGN‐073 facilitator exhibited a massive decrease in total deposited amyloid in their cortices, the total number of plaques, and plaque densities, with significant differences compared to both untreated and TGN‐020‐treated APPPS1 mice. In another study utilizing the AQP4 facilitator on 5xFAD mice, a daily gavage of 400 mg/kg for 3 months decreased insoluble Aβ40 and Aβ42 within the cortex and hippocampus.^56^ Moreover, AQP4 deletion in the same genetic background significantly increased total amyloid accumulation in the cortex with larger compact plaques, but with a relative reduction in the number of dense‐core plaques compared to controls, indicating an altered amyloid accumulation pattern in the absence of AQP4.^57^ Although the total plaque areas, diameters, and densities showed a clear‐cut drop for the facilitator‐treated animals in our experiments that was paralleled by a steep increase for the inhibitor‐treated animals, notably, the average deposit densities showed no difference between untreated APPPS1 and APPPS1 facilitator‐treated animals, suggesting little change in the degree of compactness after AQP4 facilitation despite large differences in the total plaque burden. That Aβ42 is predominantly produced in APPPS1 animals and that AQP4‐mediated drainage clears predominantly Aβ40 suggest why APPPS1 facilitator‐treated mice show even smaller Aβ40/Aβ42 ratios compared to untreated APPPS1 mice. A further decrease in the Aβ40/Aβ42 ratio for the inhibitor‐treated animals explains a massive increase in Aβ42, both as absolute value and relative to the Aβ40, which results from blocking IPAD. This, together with larger plaques for inhibitor‐treated animals, suggests that the neuropil is saturated with soluble/insoluble Aβ that cannot diffuse toward the IPAD and, therefore, precipitates and forms larger plaques with dense cores and large diffuse coronas, a pattern that best describes inhibitor‐treated APPPS1 mice (Figure 8).

We further performed a morphological plaque–vessel association analysis to explore the vasculotropic plaque formation hypothesis.^16^, ^17^ We showed that both inhibitor‐ and facilitator‐treated mice exhibited decreased frequencies of plaques associated with vessels compared to untreated APPPS1 animals; however, it was the facilitator‐treated model that harbored a massive decrease. Therefore, it is conceivable that extreme mechanisms at the opposite ends of the same physiopathological spectrum are responsible for this common morphological pattern. Thus, on the one hand, while the APPPS1 mouse model overproduces mutant human APP/Aβ^36^ that already overwhelms the clearance pathways, a further inhibition of IPAD cannot produce a significant number of new plaques developing around vessels. On the other hand, facilitating IPAD against this Aβ overproduction background favors its clearance, which leaves mostly less‐diffusible Aβ42,^58^ promoting plaque formation around neurons. This hypothesis fits well with the decreased Aβ40/Aβ42 ratio observed in facilitator‐treated animals compared to untreated APPPS1 mice. However, it was the inhibitor‐treated group that showed the most significant increase in Aβ42, rather than the facilitator‐treated group as expected, suggesting that IPAD must also engage less soluble Aβ. Indeed, our data support this, as the facilitator reduced both insoluble Aβ, as shown by immunohistochemistry/ThS staining, and soluble Aβ, as revealed by Western blotting. For both APPPS1 mice and human AD, stable amyloid deposits tend to emerge first in the entorhinal cortex and hippocampus, likely due to a failure in the transport of processed APP along axonal pathways from the entorhinal cortex,^36^, ^59^, ^60^, ^61^, ^62^ and in time, intense trafficking of soluble/insoluble amyloid over the IPAD pathway leads to the obliteration of these vessels, a hypothesis that aligns perfectly well with our result of decreased plaque–vessel association in the hippocampus after AQP4 facilitation.

To the best of our knowledge, no studies have yet assessed amyloid accumulation after extended TGN‐020 treatment in WT mice; however, available neuropathological data from AQP4 knockout mice have not shown any significant neuronal/glial cell loss, loss of myelin, or ultrastructural changes to the BBB,^63^, ^64^ suggesting that amyloid depositions in the neuropil or fibrillar material around the BBB are unlikely to occur. Another contributing factor is that WT aged rodents do not spontaneously develop amyloid plaques, as their endogenous Aβ does not aggregate into amyloid due to three different amino acid residues within their Aβ peptide compared to human Aβ,^65^ and this is also why presenilin FAD mutations in mice do not result in amyloid deposits.^66^

We have also evaluated anxiety levels and memory performances in WT C57BL6 mice and untreated APPPS1, APPPS1‐TGN‐020, and APPPS1‐TGN‐073 animals. Although untreated APPPS1 animals tended to perform worse than WT animals, these differences were not statistically significant until the age of 3 months. This is in line with previous studies on the APPPS1 model, where cognitive deficits in spatial learning and memory emerged around 7 months of age.^67^ Our mice were significantly younger than the reported age for behavioral changes,^67^ yet the AQP4 inhibitor and facilitator treatments induced significant effects at this early stage. The AQP4 inhibitor, in particular, reduced anxiety levels with measurements, well below those of both WT and untreated APPPS1 animals, and this effect was evident at both 2 and 4 weeks of treatment. Similarly, human *post mortem* studies have shown reduced AQP4 expression in brains of patients with depression, some suggesting that AQP4 autoantibodies might contribute to an immune‐mediated mechanism.^68^

The AQP4 facilitator, on the other hand, improved anxiety level/memory performance at both time points, bringing these metrics closer to WT levels. While it is known that AQP4 deletion amplifies cognitive deficits in 12‐month‐old APPPS1 mice by increasing Aβ accumulation,^69^ it has not been clear until now whether pharmacological inhibition can have the same effect in a short amount of time and whether AQP4 facilitation can rescue the APPPS1 phenotype and restore the behavioral pattern of the WT group.

One of the limitations of our study is the lack of statistical power to demonstrate significant differences in brain regions that show a lower burden and high variability in amyloid deposition. However, our ability to identify significant differences in cortices and other key brain areas relevant for AD suggests that the trends observed in these regions might reach significance if a larger animal size were studied. Additionally, while we report the substantial effects of the AQP4 inhibitor on increased Aβ accumulation and accelerated behavioral deficits, it is important to note that TGN‐020 has also been shown to “partially” target AQP1, another water channel present in the brain blood vessels.^28^, ^70^, ^71^

## 5 CONCLUSIONS

This is the first study to directly compare the effect of AQP inhibition and facilitation within the same APPPS1 background, focusing on the quantality and quality of amyloid deposits, as well as their impact on functional memory and anxiety in these animals. We used an APPPS1 model rather than a more complex tau/APP model, as our focus here was on understanding amyloid clearance via IPAD, and passive gradient‐driven diffusion is already complicated by the involvement of insoluble fractions apparently co‐cleared along the IPAD pathway. Future investigations of both soluble and insoluble Aβ/tau in animal models would represent an essential direction for elucidating the pathophysiology of AD and related proteinopathies.

## AUTHOR CONTRIBUTIONS

Daniel Pirici, Samir Kumar‐Singh, and Bogdan Catalin made substantial contributions to the design of the study and prepared the draft of the manuscript. Animal treatments and behavior analysis were performed by Bogdan Catalin, Ianis Kevyn Stefan Boboc, and Ilona Mihaela Liliac. Histopathology work, immunohistochemistry, and image analysis were performed by Daniel Pirici, Marina Daniela Manescu, Valentin Octavian Mateescu, Gabriela Camelia Rosu, Anca‐Maria Istrate‐Ofiteru, and Costin Teodor Streba. Ioana Baldea performed Western blotting analysis. Costin Teodor Streba reviewed the statistical data analysis. Samir Kumar‐Singh and Daniel Pirici reviewed the manuscript and gave final approval of the version to be published. All authors read and approved the final manuscript.

## CONFLICT OF INTEREST STATEMENT

The authors declare no competing interests. Author disclosures are available in the Supporting Information.

## CONSENT STATEMENT

Not applicable (no human subjects involved).

## Supporting information



Supporting Information

Supporting Information

Supporting Information
