# Supplementary material for: Aquaporin 4 modulation drives amyloid burden and cognitive abilities in an APPPS1 mouse model of Alzheimer's disease
Source: Alzheimers Dement. 2025 May 6;21(5):e70164. doi: 10.1002/alz.70164 (PMC12056304; doi:10.1002/alz.70164)
Supplement: Supplementary file 1 — Supporting Information [file ALZ-21-e70164-s001.docx]

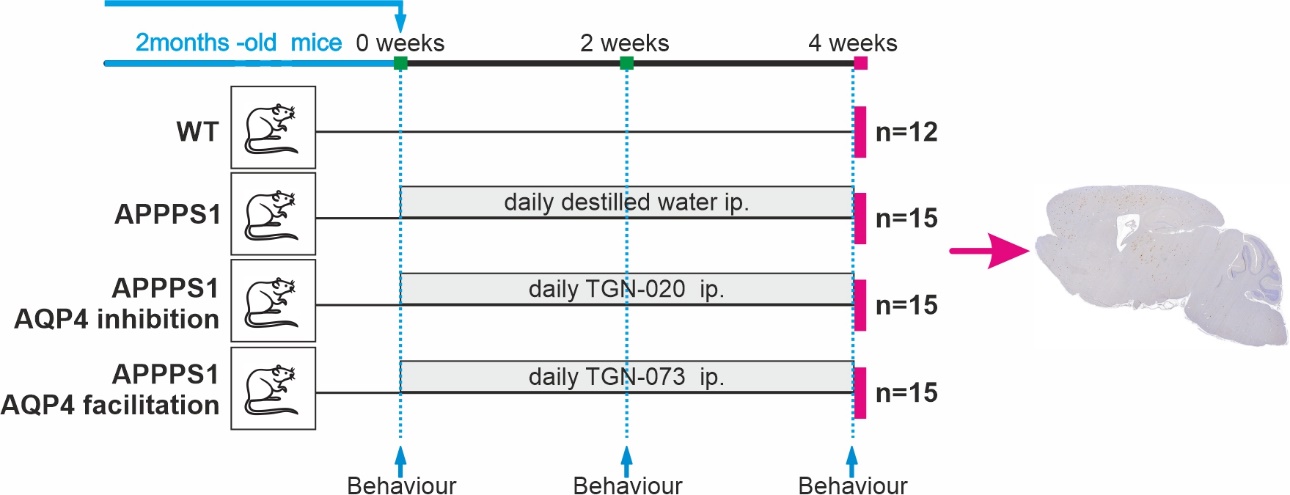


**Supplementary Figure 1** Two-months old APPPS1 mice were administrated intraperitoneally with either TGN-020 AQP4 inhibitor (200mg/kg), TGN-073 AQP4 facilitator (200mg/kg) or destilled water daily for 1 month. An untreated age-matched wild-type C57BL/6J mice group was followed-up without any treatment. Anxiety levels and memory performance were evaluated at the beginning and at 2 and 4 weeks of the treatment, after which animals were euthanized and brains harvested and studied morphometrically for plaque burden and plaque-vessel relationships.
