# Supplementary material for: Aquaporin 4 modulation drives amyloid burden and cognitive abilities in an APPPS1 mouse model of Alzheimer's disease
Source: Alzheimers Dement. 2025 May 6;21(5):e70164. doi: 10.1002/alz.70164 (PMC12056304; doi:10.1002/alz.70164)
Supplement: Supplementary file 2 — Supporting Information [file ALZ-21-e70164-s002.docx]

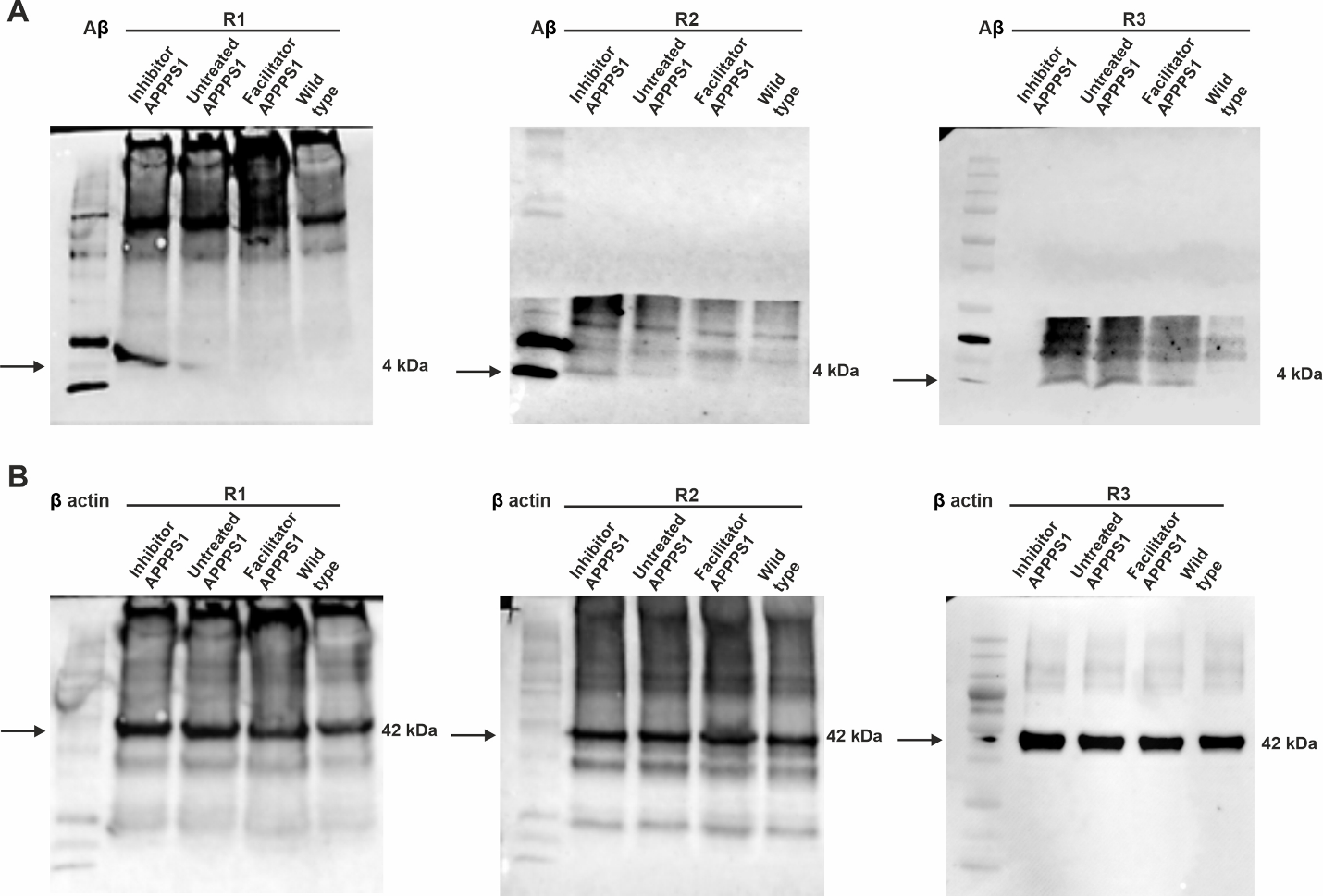


**Supplementary Figure 2** Western Blot images from three independent replicates (R1, R2, R3), shwon side by side: amyloid β peptide (Aβ) (**A, B**), and β-actin quantification for each membrane (**C, D**).
